# Effect of various irrigation protocols and antioxidant application on bonding performance of two adhesive systems to coronal dentin

**DOI:** 10.4317/jced.61303

**Published:** 2024-04-01

**Authors:** Nada Omar, Haidy N. Salem, Ahmed Abdou, Lamiaa M. Moharam

**Affiliations:** 1 Restorative and Dental Materials Department, National Research Centre, Giza, Egypt; 2 School of Dentistry, Newgiza University, Giza, Egypt; 3 Faculty of Dentistry, Al-Ayen University, Thi-Qar, Iraq

## Abstract

**Background:**

The aim was to evaluate the effect of different irrigation protocols and antioxidants application on the shear bond strength (SBS) of two different adhesive systems to deep coronal dentin at different temperature.

**Material and Methods:**

One hundred and twenty human premolar teeth were cut longitudinally in two halves, then the prepared specimens were allocated into four main groups according to the irrigation protocols; Distilled water (control), Sodium hypochlorite (NaOCl) + Ethylenediamine Tetra-acetic Acid (EDTA), and NaOCl + Editronate (HEDP), two subgroups according to irrigation solutions temperature; 4°C and 37°C, two divisions according to grape seed extract (GSE) application (with and without), and two subdivisions according to the applied adhesive systems; OptiBond-All-In-One (OPT) and ScotchBond Universal (SBU). Adhesives were applied in self-etch (SE) mode and resin composite material discs were built. Specimens were kept in distilled water for 24-h at 37°C before SBS testing. Four-way ANOVA and Tukey HSD tests were used for data analysis (α=0.05).

**Results:**

4°C irrigants solution temperature showed the least significant SBS values, distilled water group showed significantly higher SBS values compared to NaOCl + EDTA and NaOCl + HEDP groups respectively. GSE application improved dentin bond strength significantly within each adhesive.

**Conclusions:**

Increasing the temperature of the irrigation solutions has significantly enhanced the SBS of coronal dentin. Antioxidants application following different irrigation protocols has improved the SBS.

** Key words:**Irrigation protocols, temperature, bond strength, coronal dentin, antioxidant, adhesive systems.

## Introduction

Successful endodontic treatment plays a fundamental role in alleviating the patients’ pain and discomfort as well as extending the life span and maintaining the function of the teeth. Root canal irrigating in endodontics is a critical step that cannot be spared. Root canal irrigants have chemical and physical consequences essential for proper disinfection and debridement of the root canals. Different irrigating solutions were proposed to ensure optimal removal of dentin chips, pulpal tissues residues, and microorganisms via a flushing process throughout and next to root canal instrumentation. More than a few root canal irrigants can dissolve the inorganic and/or organic tissues inside the root canal, while others have an antimicrobial effect destroying the microorganisms upon direct contact (1). A mixture of irrigants in suiTable combination could enhance the process of root canal cleaning and lead to successful consequences (2).

Root canal irrigants can be categorized by their mode of action into bactericidal, non-bactericidal, chelating, and herbal (2). Sodium hypochlorite (NaOCl) is a common irrigating solution, due to its organic tissue dissolving capability and wide spectrum antimicrobic action (1). Some factors could enhance tissue dissolving by NaOCl, such as pH rising, ultrasound agitation, increased working time, and the temperature solution and its concentration (1). NaOCl has low molecular weight enabling its diffusion inside hydroxyapatite-encapsulated collagen matrix, thus denaturing the collagen, oxidizing its organic matrix, while negatively altering dentin mechanical properties (3). Consequently, using a combination of inorganic and organic tissue-dissolving irrigating solutions was suggested (2). An ideal root canal irrigation could be established via combination of two or more irrigants, in a definite order for an effective and reliable irrigation process. EDTA is a demineralizing agent which can effectively eliminate the smear layer and increase the patent dentinal tubules diameter (2). Weak chelating agents such as Etidronic acid (1-hydroxyethylidene-1,1-disphosphonate “HEDP”) has been recently presented as a counterpart of EDTA, for more simplification and reduction of the time required for proper root canal irrigation. HEDP was reported to have the least effect on dentin surface, though it is still able to decrease the smear layer (4). Nonetheless, HEDP is not recommended to be applied alone as a final root canal irrigant due to its inadequate decalcifying action. Hence, it was suggested to mix HEDP with NaOCl solution for more competent root canal irrigation that combines the effective features of both irrigants (5). The temperature of the irrigating solutions plays a significant role in endodontic treatments. Preheating root canal irrigants might decrease surface tension of the irrigating solution causing improved diffusion of the irrigants which leads to competent smear layer eradication (6), that significantly influences the bond strength of the dentin. It was reported that the temperature of EDTA final irrigant affects the bond strength to the dentin substrate (7). Using natural antioxidants is highly increasing as an attempt to reverse the compromised collagen fibrils that happened due to root canal irrigation, as they can restore the redox potential of the previously oxidized dentin surface resulting in an adequate polymerization of the restoration system as a whole (8). Treatment with natural antioxidants demonstrated acceptable outcomes in restoring the depleted dentin bond strength after using different irrigation solutions immediately after RCT and before the application of the adhesive systems and final restorations (8). GSE is a potent antioxidant agent that is mainly composed of proanthocyanidin (PA). It can eliminate the smear layer, has significant antibacterial properties, and enhance the mechanical properties of dentin. PA is responsible for cross-linking of the collagen molecules within the dentin by increasing the number of intra- and inter-molecular collagen cross-links, hence reinforcing the collagen matrix of dentin and enhancing the constancy of the collagen by avoiding enzymatic degradation and forming more sTable collagen. Hence, maintaining the mineral constituent for improved microhardness and bond strength to dentin (9). While there are a lot of research assessing the consequences of NaOCl and EDTA irrigation on coronal dentin bond strength, the literature that discuss the outcome of HEDP irrigation on coronal dentin bond strength are quite limited and the effect of HEDP on composite resin’s bond strength to dentin needs more research. Therefore, it is important to determine the efficacy of different irrigation protocols and antioxidant application on the SBS of deep coronal dentin using two different adhesive systems at two different temperatures.

The null hypotheses tested were (a) the different irrigation protocols had no effect on the bond strength to deep coronal dentin using different adhesive systems at different temperatures with and without antioxidant application. (b) the bond strength to deep coronal dentin had not been influenced by the application of the antioxidant agent following different irrigation protocols application at different temperatures using various adhesive systems. (c) the temperature of the irrigating solutions had no effect on the bond strength to deep coronal dentin with and without the application of the antioxidant agent following different irrigation protocols application using different adhesive systems. (d) the adhesive type had no impact on the bond strength to deep coronal dentin with and without the application of the antioxidant agent following different irrigation protocols application at different temperatures.

## Material and Methods

-Selected Materials

Three root canal irrigating solutions; (Distilled water, NaOCl+EDTA, and NaOCl+HEDP), two adhesive systems; (SE adhesive; OptiBond-All-In-One (OPT), and universal adhesive; Scotchbond Universal (SBU)), one nanohybrid resin composite restorative material (Filtek™ Z350 XT Universal), one prepared GSE gel (10% concentration), were used in this study. The materials brand name, description, composition, and their manufacturers are registered in Table 1.

**Table 1:**
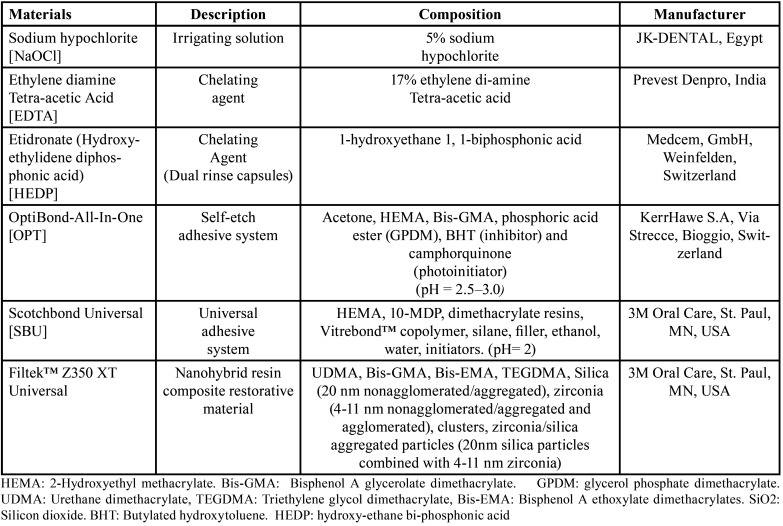
The materials used in the study and their composition, description, and manufacturer.

-Preparation of GSE antioxidant agent gel

An amount of 0.25 mg of GSE powder (NOW Foods, Grape Seed Standardized Extract, Bloomingdale, IL, USA) were dissolved in 2.5 ml of purified water at room temperature, to attain a solution of GSE of 10% concentration. The obtained solution was additionally stirred in a magnetic stirrer for 5-min to guarantee thorough dissolving of the powder into the purified water. Next, Carbopol® resin powder (Carbomer 940, Sanarelab LTD, KH, London, GB) was prepared by moderate mixing in the previously prepared 10% GSE solution. The mix was stirred using the magnetic stirrer until thickening. Subsequently, the thickened mix was neutralized by adding thriethanolamine PioChem, Giza, Egypt) drops until a transparent gel was obtained. The thriethanolamine amount was adjusted to acquire a final gel of pH=7 (10).

-Teeth selection 

Sound human premolar teeth extracted for orthodontic purposes were collected then calculus debris and soft tissues remnants were removed using a sharp hand scaler. The teeth were inspected using a magnifying lens of x25 magnification to eliminate fractured, defective, or cracked teeth. The teeth were kept in 0.1% thymol solution at 4°C for up to 3-m after extraction. The solution was replaced with a fresh one once per week until use .

-Tooth specimens’ preparation

A double-side cutting diamond disc mounted to a low-speed handpiece was used to cut the roots of the premolar teeth 2-mm below the cementoenamel junction. Barbed broaches were utilized to remove the pulp tissues. Each tooth was cut parallel to its long axis using a precision sectioning saw machine (IsoMet 1000, Buëhler, Lake Bluff, IL, USA). To obtain a standardized deep dentin, the dentin surfaces were ground under wet conditions using a 400-grit silicon carbide paper (SiC) to acquire a flat and smooth dentin surface (11). Then 600-grit SiC papers were employed for 1-min in a circular motion to obtain a uniform and standardized smear layer (11,12). Specimens were checked using a stereomicroscope (Olympus® BX 60, Olympus Optical Co. LTD, Tokyo, Japan) to eliminate any defects. Then the specimens were firmly embedded in chemical cure acrylic resin blocks. Next, specimens were instantaneously stored in distilled water after complete polymerization of the acrylic resin.

-Experimental design of the study

**Figure 1:**
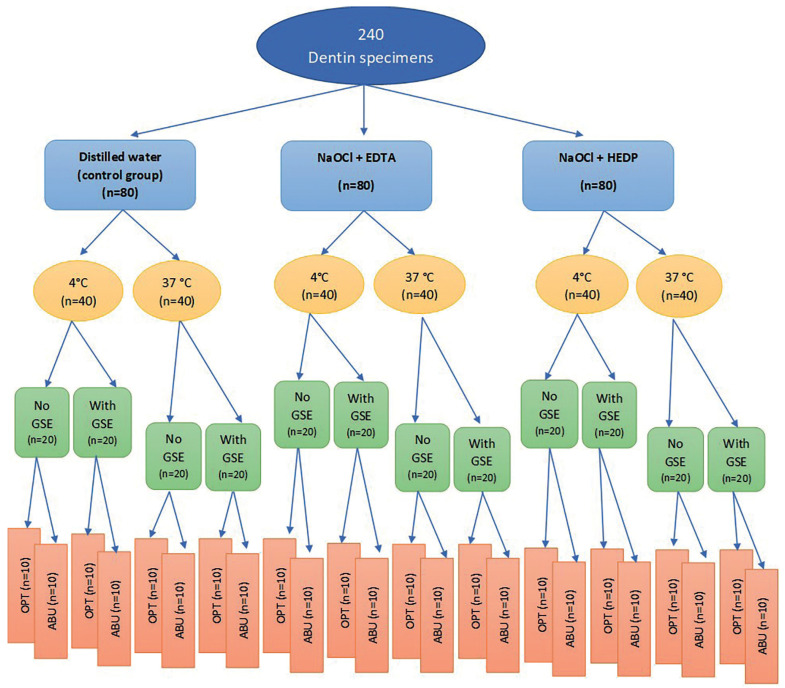
Flowchart showing the experimental design and groups of the study.

One hundred and twenty premolar teeth were sectioned into two halves to obtain a total of 240 specimens. The specimens were randomly apportioned into three main groups (n=80), representing the three irrigation protocols (Distilled water (control group), NaOCl + EDTA and NaOCl + HEDP). Each main group was further divided into two subgroups (n=40) conferring the temperature of the irrigating solutions (4°C and 37°C). Then each subgroup was divided into two divisions (n=20) representing the antioxidant application (with and without GSE application). Each division was further divided into two subdivisions (n=10) according to the two adhesive systems used in the study (OPT and SBU). The specimens grouping, frequency and study design are presented in Fig. 1.

-Application of the irrigation protocols 

The assigned specimens were placed in clear polyethylene plastic containers in 5 ml of 17% EDTA solution for 1-min followed by immersion in 10 ml of 5% NaOCl solution for 2-min at either 4°C or 37°C, and then rinsed with water for 2-min. Meanwhile, other specimens’ groups were put in polyethylene plastic containers then immersed in 5 ml of equal amounts of 5% NaOCl solution mixed with HEDP at either 4oC or 37oC for 10-min, and then rinsed with water for 2-min (13). The control groups were immersed in distilled water for 2-min and then rinsed with water for 2-min at either 4°C or 37°C.

-Application of the GSE antioxidant agent gel

After the application of different irrigation protocols, 10% GSE gel was applied to the treated coronal dentin surfaces of the assigned specimens, using micro brushes, and then left uninterrupted on the dentin surfaces for 10-min (14). The gel was removed using cotton rolls then the specimens were rinsed under tab water for 30-s (15).

-Application of the adhesive systems 

OPT and SBU adhesives were applied to the assigned deep coronal dentin specimens using SE technique according to their manufacturers’ commands. Two to three coats of both adhesives were actively applied for 20-s to the treated surfaces using micro brushes followed by mild air streaming for 5-s until adhesives were no longer moving, and the solvent had entirely evaporated (12). Next, each adhesive was photo-cured for 10-s using light-emitting diode (LED) curing unit (Elipar S 10, 3M ESPE, USA). The output intensity of LED curing unit was measured (≥ 1000/cm2) and checked intermittently with porTable radiometer (Demetron 100, Kerr Corporation, Orange, CA, USA). Clear plastic rings (2-mm height x 1.8-mm diameter) were positioned just above the pulp chamber boundaries at deep coronal dentin. Z350 XT Universal resin composite material was inserted in a single increment inside the clear plastic rings. Transparent celluloid strips were positioned on the top of the restorations. Then the resin composite was light cured for 20-s conferring to the manufacturer commands. The plastic rings were cut using sharp blades and the excess resin composite flashes extending past the base of the composite discs were removed. Lastly, the specimens were reserved in tight-seal polyethylene containers in distilled water at 37°C for 24-h until SBS test was conducted (16).

-Shear bond strength test (SBS)

A metallic bladed chisel attachment was mounted at the upper jig of the universal testing machine (Instron®, Model 3345, Instron Instruments, Buckinghamshire, UK). The chesil was situated as close as possible to the dentin-restoration interface. The specimens’ blocks were fixed to the lower jig of the machine, and the test was run at 0.5 mm/min cross head speed till specimens’ failure. The maximum force was calculated in MPa. SBS was calculated using the machine computer software (BlueHill® Universal, Instron Testing Software, Buckinghamshire, UK).

-Mode of fracture analysis

Debonded specimens were examined using stereomicroscope at magnification x35. Fracture modes were identified as adhesive when the identified failure occurred at the composite/tooth interface, cohesive when the detected failure occurred within the resin composite or dentin, and mixed when adhesive and cohesive failure were both detected.

-Statistical analysis 

Data checked for normality using Kolmogorov-Smirnov test. SBS showed normal distribution, so four-Way ANOVA was used to show the effect of irrigation solution (NaOCl + EDTA, NaOCl + HEDP, and Distilled water), temperature (37°C, and 4°C), adhesive type (SBU, and OPT), and GSE application (no GSE, and GSE application) on the mean SBS. Four-way ANOVA was used to compare between tested groups and irrigation steps. Tukey’s HSD post hoc test used for multiple comparison between subgroups. Significance level was set at *p*=0.05. Statistical analysis was performed using IBM SPSS Statistics Version 20 for Windows (IBM SPSS, version 26, Armonk, NY, USA).

## Results

**Figure 2:**
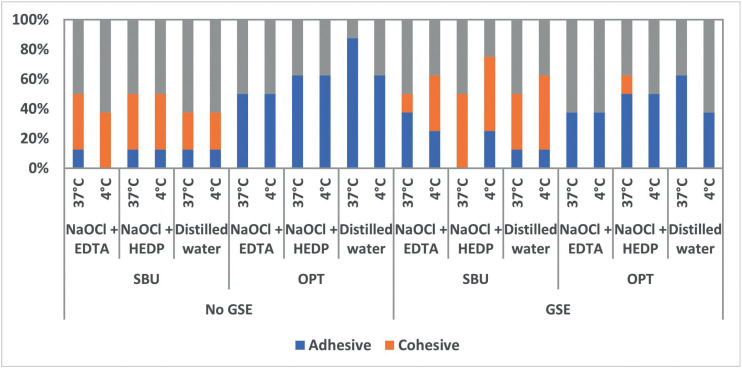
Showing the mode of fracture of the different groups of the study.

Table 2 and Table 3 showed the results of SBS. Four-way ANOVA test showed that all variables had a significant effect on mean SBS (All, *p*<0.001) and the interaction between the variable also showed a significant effect on SBS at *p*=0.003. For the different tested irrigants, 4°C showed the least SBS compared to 37°C for the tested adhesives and GSE application. While for distilled water, 4°C showed the least SBS values compared to 37°C only for SBU with no GSE application. For the comparison between the different irrigants and the control group, the distilled water group showed significantly higher SBS values compared to both NaOCl + EDTA and NaOCl + HEDP groups within each adhesive, temperature, and GSE application. NaOCl + HEDP group showed the least SBS values compared to NaOCl + EDTA groups within each adhesive, temperature, and GSE application. GSE application improved dentin bond strength significantly within each adhesive. ‘SBU’ universal adhesive showed a significantly higher SBS values when compared to the SE adhesive ‘OPT’ with and without GSE application. Fig. 2 demonstrated the failure mode analysis, and the results showed that cohesive failure was shown only for SBU adhesive bonded groups while the predominant failure mode type for OPT adhesive bonded groups were adhesive failure regardless of GSE application, irrigation type and/or the temperature of the irrigation solution.

**Table 2:**
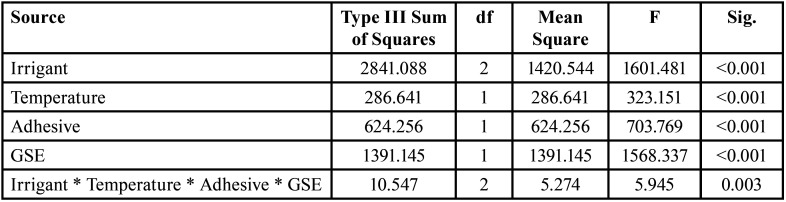
Four-Way ANOVA showing the interaction between the different variables of the study and their effect on the mean SBS.

**Table 3:**
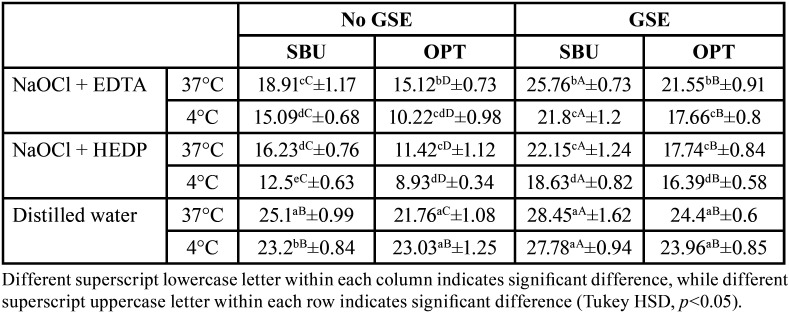
The effect of the different variables of the study on the SBS of coronal dentin.

## Discussion

Current research tested the effect of different irrigation protocols at different temperatures and antioxidant application on SBS of two adhesives to coronal dentin. The results revealed a statistically significant effect of the different irrigation protocols on SBS. Therefore, the first null hypothesis was rejected. Different irrigation solutions have a significant impact on the final bond strength to dentin (5), which was in accordance with our results. Successful endodontic therapy offers proper chemo-mechanical debridement for root canals. Moreover, ultimate endodontic irrigation solution should dissolve necrotic pulp tissues, have an antimicrobial action, have decreased cytotoxicity, and eliminate smear layer (5). The most widely used irrigants during the root canal treatment is NaOCl and EDTA to ensure complete elimination of the smear layer.

Present study displayed that consecutive application of EDTA and NaOCl irrigation caused a significant reduction in the adhesion of both adhesive systems compared to the control group with distilled water. This might be due to demineralization of dentin by 17% EDTA application, that is a debilitated acid etching representative along with consequent dentin deproteinization executed by NaOCl. It was reported that NaOCl decomposes resulting in free oxygen production preventing the resin polymerization and thus the bond strength is significantly compromised (3). This was in accordance with Garcia *et al*. (17) who displayed that successive application of EDTA and NaOCl irrigants showed bond strength values close to the control group yet significantly lower. They owed such findings to the effect of NaOCl that might have dissolved the organic components eliminating the smear layer. Moreover, it was concluded that NaOCl was responsible for degeneration of root dentin via organic content dissolution, specifically type I collagen fibrils dissolution (18). Thus, destructing the collagen network integrity, which plays a significant role in the adhesion mechanism (17,18). Meanwhile, EDTA demineralizes the inorganic component of the dentin and dissolves the smear layer. Such a combination might have developed progressive demineralization of coronal dentin surface and widening of the dentinal tubules’ orifices (13) which might be responsible for impairing the overall bond strength with both adhesive systems. Reduction in the coronal dentin bond strength of EDTA + NaOCl groups could be owed to presence of some NaOCl irrigation solution residues that might have penetrated the patent dentin tubules, hence interacting with the successive adhesive system affecting its polymerization leading to a significant reduction in the final dentin bond strength (19). Nonetheless, EDTA is an effective chelating agent with a potent demineralizing influence, that might soften the dentin thus enlarging the dentinal tubules and denaturing collagen fibrils. It was reported that EDTA might lead to dentin surface erosion upon increasing its time of application (19), and consequently EDTA irrigant may negatively affect dentin bond quality and marginal sealing of the final restoration (5). Etidronic acid solution is not recommended to be used alone, as it should be mixed with NaOCl upon root canal preparation, to make a good use of the specific aspects of each constituent (4). The results of the current study revealed that NaOCl + HEDP groups showed the least bond strength values. This agreed with Arslan *et al*. 2019 (5) who suggested that HEDP could be responsible for the development of more demineralized dentin zone at the resin-dentin interface due to its chelating action, thus negatively affecting the final dentin bond strength. Such finding was confirmed by the eminent number of the adhesive fractures at dentin/resin interface in NaOCl + HEDP and NaOCl + EDTA groups correspondingly compared to control group at the different temperature for both adhesive systems regardless the application of GSE.

Free radicals released during the procedure of pulp tissues dissolution were found to compete with those created during light curing of resinous restorations. Thus, leaving the end of the monomer chain opened with inadequate resin polymerization, and therefore putting the bond strength of the adhesive system at risk. Additionally, calcium and phosphate contents of dentin are much decreased, and the mechanical properties of dentin like the hardness, flexural strength and modulus of elasticity are deteriorated. Hereafter, the micro-mechanical interactions between adhesive monomer and the dentin would decline after irrigation with NaOCl (20). To counteract the detrimental effect of the irrigation solutions on dentin, an antioxidant solution application has been advocated previous to the application of the adhesive system (21). Antioxidant agents are able to interact with the by-products produced by NaOCl dissociation causing neutralization and counteract the oxidizing effect of NaOCl on the treated dentin surfaces (22) thus, ensuring success and longevity of the final coronal restoration. In this regard, the results of the current study showed that the application of GSE, which is a powerful antioxidant agent, has a statistically significant effect on SBS of coronal dentin following different irrigation protocols. Thus, the second null hypothesis was rejected. The increased SBS values of GSE groups compared to groups with no GSE treatment could be owed to GSE interaction with the residual oxygen released by the irrigants groups containing NaOCl solution, which is accountable for impeding the polymerization of the adhesive system and the successive resin composite, therefore improving the bonding performance of the final restoration (21). This finding agreed with Gascón *et al*. 2023 (21) and Kumar *et al*. 2019 (22) who concluded that the final irrigation with antioxidants such as PA and bamboo salt was able to eliminate the hazards of bond strength deterioration to dentin following the application of NaOCl irrigation solution. Cross-linking ability of PAs to dentinal collagen might be an additional probability for the enhanced bond strength. The collagen’s proline-rich proteins were reported to be highly attracted to PAs, and forming strong hydrogen bonds with them, leading to high inter- and intra-molecular crosslinking (23). Thus, helping in stabilization of the collagen, which became exposed during irrigation of the root canal using EDTA chelating agent. In this basis, Kalra *et al*. 2013 (24) studied the effect of different PA-based antioxidant agents on the bond strength and biodegradation resistance of the demineralized root canal dentin. They concluded that PAs application following final canals irrigation has improved biodegradation resistance of dentin, preserving the seal at the resin sealer-root dentin interface. However, this finding was opposed by Stevens in 2014 (25) who demonstrated that the development of an adhesive bond strength with a minimum of 50% of the original bond strength subsequent to rinsing with 10% sodium ascorbate was not enough to be effective. This finding was additionally established by the elevated number of the adhesive fractures at the dentin/resin interface in NaOCl-containing groups compared to control group using distilled water regardless the different temperature and the adhesive systems.

Clinically, proper management of pain during the endodontic processes and postoperative phases is one of the utmost essential objectives of dental clinicians. Keskin *et al*. 2017 (26) assessed the effect of application of irrigating solutions with low temperature on the post-operative pain in teeth with irreversible pulpitis subsequent to single-visit RCT. They reported that the post operative pain and inflammation were significantly reduced. Furthermore, the temperature of irrigating solutions during RCT could be decreased to a range of 1.5 to 4°C to reduce the post-operative pain clinically using a refrigerator to acquire cold solutions with a thermometer for temperature control (27). Al-Nahlawi *et al*. 2016 (28) concluded that using irrigant solutions at 4°C could successfully eliminate the post-endodontic pain clinically. Therefore, the root canal irrigants used in the current study were tested at a low temperature of 4°C and compared to 37°C irrigants temperature. Likewise, the temperature of the final irrigant was reported to affect the bond strength to the dentin substrate. The results of the study revealed that the temperature of the irrigation solution had a statistically significant consequence on the bond strength to coronal dentin. Subsequently, the third null hypothesis was rejected. Temperature of the irrigation solutions at 37°C showed a significant increase in the bond strength values compared to 4°C. This could be owed to the increased ability of the irrigants to remove the smear layer at elevated temperature. This finding agreed with Cunningham and Balekjian (29) who showed that increasing temperature of irrigants significantly increases its dissolving ability of organic content of smear layer and denaturation underlying dentin, thus affecting the bonding performance of the SE adhesives to coronal dentin negatively. This could be explained by increased dentin deproteinization due to increased proteolytic activity of the NaOCl on the collagen fibrils of dentin jeopardizing the final bond strength. Çiçek and Keskin (30) concluded that EDTA and MTAD application at 25°C and 37°C was more competent than application of the solutions at 4°C even at the apical dentin for the removal of smear layer. Moreover, increasing the temperature of the irrigation solutions was found to increase the removal of the smear layer and the dissolution of the pulp tissues increasing dentin bond strength (6). This outcome was manifested by the predominant number of adhesive fractures at the dentin/resin interface in NaOCl + HEDP and NaOCl + EDTA groups respectively compared to the control group using distilled water regardless of the tested adhesive systems.

Long-term success of root canal treated teeth relies upon endodontic treatment quality and the consequently used restorative technique. Furthermore, effective coronal seal promotes the success of RCTs which can be accomplished by proper bonded restorations. So as to, the functional stresses can be conveyed throughout the bonded interface to reinforce the weakened tooth structure (3). Additionally, different deproteinizing irrigants were reported to dissolve the organic component of the smear layer on the prepared dentin surfaces upon bonding using SE adhesives that can modify the smear layer integrating it into the hybrid layer, revealing acceptable bonding performance (31). This was found to decrease the organic to inorganic contents ratio on the coronal dentin surface, leading to thin smear layer formation which might enhance the adhesive monomer infiltration owed to elimination of smear layer organic contents (3). In the same context, Tay *et al*. 2006 (32) suggested that irrigation using chelating and demineralizing effect was able to develop a bonded surface similar to acid conditioning. Thus, the acid conditioning step can be avoided with SE adhesives applied after endodontic irrigation solutions. Such findings were consistent with our study. Modern SE and universal adhesives are introduced to the dental market to ensure further easy and reliable bonding procedure. Universal adhesives revealed an immediate and promising clinical bonding performance in relation to the gold standard etch-and-rinse (ER) and SE contemporary adhesives (12). Moreover, universal adhesives demonstrated significant enamel and dentin bond durability with several restorations. Consequently, two different adhesive systems (single-step SE adhesive and universal adhesive) of different chemical composition and applied in SE adhesive mode were investigated in the present study. Our results displayed that the type of adhesive system had a statistically significant effect on the SBS to the coronal dentin following the application of the different irrigation protocols at different temperatures regardless of GSE application. Accordingly, the fourth null hypothesis was rejected. It was demonstrated that resin composite restorations are the most common final coronal restorations used for restoring endodontically treated teeth (33). Moreover, the type of adhesive system plays a significant role in bonding performance of the final restoration of endodontically treated teeth. However, dentin deproteinization that happens upon exposure to NaOCl irrigation has equally influenced all types of adhesive systems. In the present study, SBU recorded higher SBS values compared to OPT for the different irrigation protocols regardless of the different temperature and GSE application. This might be owed to its chemical composition, which includes the acidic functional monomer 10-MDP (10-Methacryloyloxydecyl dihydrogen phosphate), which provides durable adhesion and better bonding performance (12). That happens via decalcification, monomer diffusion, and chemical bonding with the tooth calcium ions and the hydroxyapatite. Thus, allowing for a simultaneous double bonding with the tooth substrate in the form of conventional micromechanical as well as chemical bonding (12). The chemical bonding is accounTable for the formation of calcium salts that are created by the phosphate monomer, which are highly insoluble. According to the concept of adhesion-decalcification, the less soluble the calcium salts of an acidic molecule, the more tough and sTable the molecular adhesion. Therefore, more significant stability in an aqueous medium is evident (34). The 10-MDP acidic functional monomer is a constituent of the tested SBU adhesive monomer while SE adhesive OPT lacks such functional monomer. On the other hand, OPT is composed of GMDP (glycerol phosphate dimethacrylate) functional monomer, thus it is incapable of chemical bonding with dentin (20). GMDP interacts with inorganic components of the tooth structure forming GMPD-calcium salts that are more liable to degradation by water compared to the insoluble MDP-calcium salts formed by chemical adhesion of SBU and dentin (12). That might elucidate the lower bond strength values of OPT. Subsequently, superior SBS were observed for ‘SBU’ groups even after the application of NaOCl irrigation solution. This could be related to formation of insoluble MDP-calcium salts, which demonstrate significant resistance to hydrolysis degradation, excellent stability, and enhanced longevity at the bonding interface (35). This finding could be confirmed by the predominance of the cohesive failure mode that was only demonstrated for SBU groups, while OPT groups showed that the adhesive failure was the dominating mode of failure regardless of GSE application, the irrigation type and/or the tested temperature. Consequently, due to the need for placement of an immediate final restoration after endodontic treatments in most clinical cases, in which irrigation solutions that contain NaOCl were applied, adhesive systems composed of the 10-MDP functional monomer is highly proposed.

Further research is advocated to assess the effect of different irrigation protocols at different temperatures on the bonding performance, surface microhardness and fracture resistance of the different adhesive systems using different antioxidant agents.

## Conclusions

Under the limitations of the present *in vitro* study, it can be concluded that increasing the temperature of the irrigation solutions presents an effective measure to enhance the bond strength to coronal dentin. Application of the antioxidant agent GSE improved the depleted bond strength of the dentin following the application of the different irrigation protocols. Dental adhesives containing 10-MDP monomer showed better bonding performance than the adhesives which do not contain such monomer.
